# 4-Amino­phthalimide

**DOI:** 10.1107/S160053680802388X

**Published:** 2008-07-31

**Authors:** Moloy Sarkar

**Affiliations:** a Department of Chemistry, National Institute of Science Education and Research (NISER), Bhubaneswar 751005, Orissa, India

## Abstract

The mol­ecules in the title compound (systematic name: 5-aminoisoindole-1,3-dione), C_8_H_6_N_2_O_2_, are packed through N—H⋯O inter­molecular hydrogen-bonding inter­actions. Two types of hydrogen bonds are observed: one, involving the imide group, forms mol­ecular chains along the *c* axis and another two, involving the amino group, connect the mol­ecular chains.

## Related literature

For related literature, see Paul & Samanta (2007 ▶).
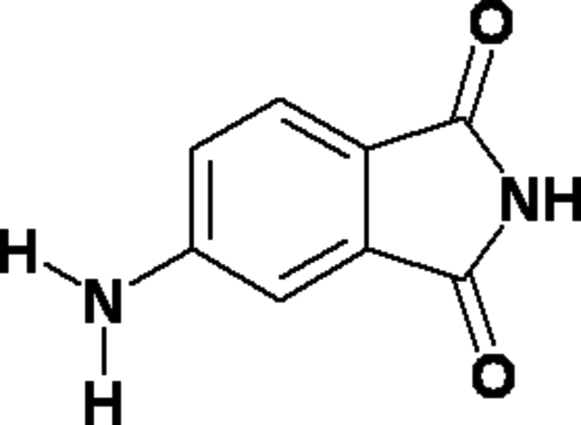


## Experimental

### 

#### Crystal data

C_8_H_6_N_2_O_2_
*M* *_r_* = 162.15
Orthorhombic, 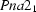

*a* = 14.5786 (19) Å
*b* = 13.0728 (17) Å
*c* = 3.7216 (5) Å
*V* = 709.27 (16) Å^3^
*Z* = 4
Mo *K*α radiation
μ = 0.11 mm^−1^
*T* = 298 K
0.25 × 0.08 × 0.06 mm

#### Data collection

Bruker SMART CCD area-detector diffractometer
Absorption correction: multi-scan (*SADABS*; Sheldrick, 2003 ▶) *T*_min_ = 0.97, *T*_max_ = 0.99
7856 measured reflections
978 independent reflections
636 reflections with *I* > 2σ(*I*)
*R*_int_ = 0.086

#### Refinement

*R*[*F* ^2^ > 2σ(*F* ^2^)] = 0.063
*wR*(*F* ^2^) = 0.126
*S* = 1.09
978 reflections
109 parameters
1 restraint
H-atom parameters constrained
Δρ_max_ = 0.22 e Å^−3^
Δρ_min_ = −0.17 e Å^−3^

### 

Data collection: *SMART* (Bruker, 1997 ▶); cell refinement: *SAINT* (Bruker, 1997 ▶); data reduction: *SAINT*; program(s) used to solve structure: *SHELXS97* (Sheldrick, 2008 ▶); program(s) used to refine structure: *SHELXL97* (Sheldrick, 2008 ▶); molecular graphics: *SHELXTL* (Sheldrick, 2008 ▶); software used to prepare material for publication: *SHELXTL*.

## Supplementary Material

Crystal structure: contains datablocks global, I. DOI: 10.1107/S160053680802388X/bg2195sup1.cif

Structure factors: contains datablocks I. DOI: 10.1107/S160053680802388X/bg2195Isup2.hkl

Additional supplementary materials:  crystallographic information; 3D view; checkCIF report

## Figures and Tables

**Table 1:** Hydrogen-bond geometry (Å, °)

*D*—H⋯*A*	*D*—H	H⋯*A*	*D*⋯*A*	*D*—H⋯*A*
N1—H1⋯O1^i^	0.86	2.09	2.924 (4)	164
N2—H2*B*⋯O1^ii^	0.86	2.28	3.122 (5)	167
N2—H2*A*⋯O2^iii^	0.86	2.17	2.996 (4)	161

Symmetry codes: (i) 
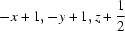
; (ii) 
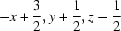
; (iii) 
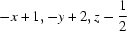
.

**Fig. 1.**
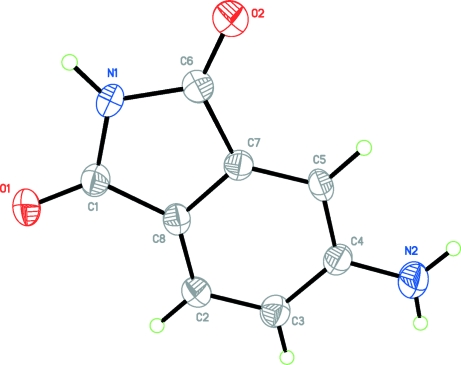
The molecular structure of the title compound with the atom-numbering scheme. Displacement ellipsoids are drawn at the 50% probability level for non-H atoms.

**Fig. 2.**
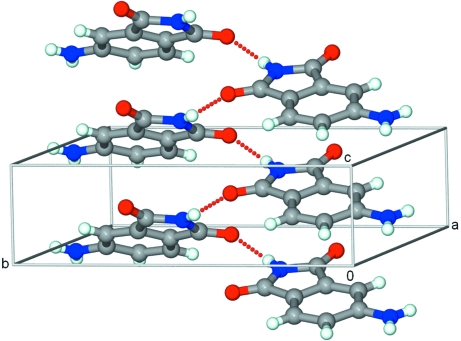
Molecular chain along the c axis.

**Fig. 3.**
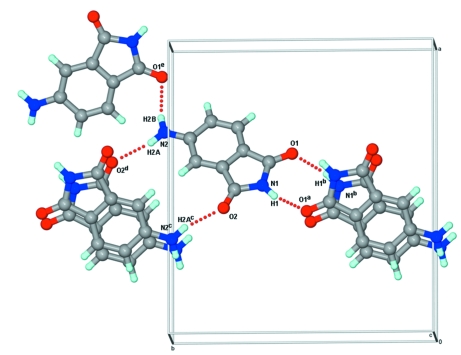
Packing diagram showing N—H···O intermolecular hydrogen bonds. [(1) N1—H1···O1a, (2) N1b– H1b···O1, (3) N2c—H2Ac···O2, (4) N2 –H2A···O2d, (5) N2—H2B···O1e; symmetry codes: (a) 1 - x, 1 - y, 1/2 + z; (b) 1 - x, 1 - y, -1/2 + z; (c) 1 - x, 2 - y, 1/2 + z; (d) 1 - x, 2 - y, -1/2 + z; (e) 3/2 - x, 1/2 + y, -1/2 + z; (1) and (2), (3) and (4) are symmetry related hydrogen bonds].

## supplementary crystallographic information

### Comment

Fluorescent electron donor-acceptor (EDA) systems, 4-aminophthalimide and its derivatives in particular, are found to be attractive candidates for the study of various photophysical processes both in conventional and non-conventional media. Very recently, 4-aminophthalimide has been used in order to investigate specific hydrogen bonding interactions in the solvation and rotational dynamics in room temperature ionic liquids (Paul and Samanta, 2007). Since the ground state structure also influences considerably the photophysical properties of the EDA molecules, we have determined the crystal structure of the title compound C_8_H_6_N_2_O_2_, (I), shown in Fig. 1. We observe that the imide group forms N—H···O hydrogen bonds (Table 1) in a helical pattern to form molecular chains along *c* axis (Figure 2). The molecules in the chains are further stabilized by π-π stacking (centroid-to-centroid distance = 3.722 Å). These chains are connected through another type of N—H···O hydrogen bonds (Table 1) involving the amino hydrogen and the unused oxygen of the phthalimide group (Figure 3).

### Experimental

The title compound was purchased from Aldrich. Tiny single crystals suitable for X-ray diffraction were obtained by slow evaporation from a solution of the compound in ethanol:water (9:1).

### Refinement

All H atoms were placed geometrically at idealized positions and refined in the riding-model approximation with the follwing constraints: C–H = 0.93 Å, N–H = 0.86 Å and with *U*_iso_(H) = 1.2*U*_eq_(C),*U*_iso_(H) = 1.2*U*_eq_(N). In the abscense of any significant anomalous effect, the data set was merged.

### Crystal data

**Table:**

C_8_H_6_N_2_O_2_	*F*_000_ = 336
*M**_r_* = 162.15	*D*_x_ = 1.518 Mg m^−^^3^
Orthorhombic, *P**n**a*2_1_	Mo *K*α radiation λ = 0.71073 Å
Hall symbol: P 2c -2n	Cell parameters from 704 reflections
*a* = 14.5786 (19) Å	θ = 2.8–18.3º
*b* = 13.0728 (17) Å	µ = 0.11 mm^−^^1^
*c* = 3.7216 (5) Å	*T* = 298 K
*V* = 709.27 (16) Å^3^	Needle, yellow
*Z* = 4	0.25 × 0.08 × 0.06 mm

### Data collection

**Table:**

Bruker SMART CCD area-detector diffractometer	978 independent reflections
Radiation source: fine-focus sealed tube	636 reflections with *I* > 2σ(*I*)
Monochromator: graphite	*R*_int_ = 0.086
*T* = 298 K	θ_max_ = 28.3º
phi and ω scans	θ_min_ = 2.1º
Absorption correction: multi-scan(SADABS; Sheldrick, 2003)	*h* = −19→19
*T*_min_ = 0.97, *T*_max_ = 0.99	*k* = −17→17
7856 measured reflections	*l* = −4→4

### Refinement

**Table:**

Refinement on *F*^2^	Secondary atom site location: difference Fourier map
Least-squares matrix: full	Hydrogen site location: inferred from neighbouring sites
*R*[*F*^2^ > 2σ(*F*^2^)] = 0.063	H-atom parameters constrained
*wR*(*F*^2^) = 0.126	*w* = 1/[σ^2^(*F*_o_^2^) + (0.0542*P*)^2^ + 0.1098*P*] where *P* = (*F*_o_^2^ + 2*F*_c_^2^)/3
*S* = 1.09	(Δ/σ)_max_ < 0.001
978 reflections	Δρ_max_ = 0.22 e Å^−^^3^
109 parameters	Δρ_min_ = −0.17 e Å^−^^3^
1 restraint	Extinction correction: none
Primary atom site location: structure-invariant direct methods	

### Special details

**Table:**

Geometry. All e.s.d.'s (except the e.s.d. in the dihedral angle between two l.s. planes) are estimated using the full covariance matrix. The cell e.s.d.'s are taken into account individually in the estimation of e.s.d.'s in distances, angles and torsion angles; correlations between e.s.d.'s in cell parameters are only used when they are defined by crystal symmetry. An approximate (isotropic) treatment of cell e.s.d.'s is used for estimating e.s.d.'s involving l.s. planes.
Refinement. Refinement of *F*^2^ against ALL reflections. The weighted *R*-factor *wR* and goodness of fit *S* are based on *F*^2^, conventional *R*-factors *R* are based on *F*, with *F* set to zero for negative *F*^2^. The threshold expression of *F*^2^ > σ(*F*^2^) is used only for calculating *R*-factors(gt) *etc*. and is not relevant to the choice of reflections for refinement. *R*-factors based on *F*^2^ are statistically about twice as large as those based on *F*, and *R*- factors based on ALL data will be even larger.

### Fractional atomic coordinates and isotropic or equivalent isotropic displacement parameters (Å^2^)

**Table:**

	*x*	*y*	*z*	*U*_iso_*/*U*_eq_	
O1	0.60766 (19)	0.53133 (19)	1.0695 (10)	0.0556 (10)	
O2	0.41159 (18)	0.7846 (2)	1.4175 (11)	0.0559 (9)	
N1	0.4932 (2)	0.6407 (2)	1.2599 (11)	0.0445 (9)	
H1	0.4542	0.5954	1.3284	0.053*	
N2	0.6919 (2)	1.0180 (2)	0.8617 (13)	0.0599 (12)	
H2A	0.6551	1.0669	0.9164	0.072*	
H2B	0.7443	1.0315	0.7663	0.072*	
C1	0.5777 (3)	0.6181 (3)	1.1116 (14)	0.0411 (10)	
C2	0.7055 (3)	0.7394 (3)	0.8804 (12)	0.0400 (10)	
H2	0.7459	0.6875	0.8153	0.048*	
C3	0.7288 (3)	0.8405 (3)	0.8309 (11)	0.0423 (11)	
H3	0.7857	0.8569	0.7338	0.051*	
C4	0.6676 (3)	0.9196 (3)	0.9256 (12)	0.0386 (10)	
C5	0.5831 (3)	0.8952 (3)	1.0800 (12)	0.0359 (10)	
H5	0.5424	0.9464	1.1489	0.043*	
C6	0.4787 (3)	0.7455 (3)	1.2853 (12)	0.0402 (10)	
C7	0.5613 (2)	0.7937 (3)	1.1281 (12)	0.0346 (9)	
C8	0.6212 (3)	0.7165 (3)	1.0282 (11)	0.0350 (10)	

### Atomic displacement parameters (Å^2^)

**Table:**

	*U*^11^	*U*^22^	*U*^33^	*U*^12^	*U*^13^	*U*^23^
O1	0.054 (2)	0.0267 (15)	0.086 (3)	0.0020 (13)	−0.0039 (19)	−0.0037 (18)
O2	0.0478 (17)	0.0435 (17)	0.076 (2)	0.0027 (14)	0.0124 (18)	0.0012 (19)
N1	0.0401 (19)	0.0284 (17)	0.065 (2)	−0.0068 (15)	0.0033 (19)	0.005 (2)
N2	0.056 (2)	0.038 (2)	0.086 (3)	−0.0050 (17)	0.012 (3)	0.001 (2)
C1	0.042 (2)	0.033 (2)	0.049 (3)	−0.0026 (19)	−0.005 (2)	0.000 (2)
C2	0.039 (2)	0.037 (2)	0.044 (3)	0.0043 (17)	0.002 (2)	−0.002 (2)
C3	0.045 (2)	0.039 (2)	0.043 (3)	−0.0019 (18)	0.002 (2)	0.001 (2)
C4	0.040 (2)	0.034 (2)	0.041 (2)	−0.0048 (17)	−0.003 (2)	0.002 (2)
C5	0.041 (2)	0.025 (2)	0.041 (2)	0.0078 (16)	−0.004 (2)	−0.003 (2)
C6	0.039 (2)	0.036 (2)	0.047 (3)	0.0022 (18)	−0.002 (2)	−0.001 (2)
C7	0.035 (2)	0.033 (2)	0.036 (2)	−0.0011 (16)	−0.0067 (19)	0.0014 (19)
C8	0.040 (2)	0.030 (2)	0.035 (3)	0.0012 (18)	−0.0032 (18)	0.0008 (19)

### Geometric parameters (Å, °)

**Table:**

O1—C1	1.226 (4)	C2—C8	1.380 (5)
O2—C6	1.209 (4)	C2—H2	0.9300
N1—C1	1.381 (5)	C3—C4	1.411 (5)
N1—C6	1.389 (5)	C3—H3	0.9300
N1—H1	0.8600	C4—C5	1.396 (5)
N2—C4	1.356 (5)	C5—C7	1.377 (5)
N2—H2A	0.8600	C5—H5	0.9300
N2—H2B	0.8600	C6—C7	1.479 (5)
C1—C8	1.467 (5)	C7—C8	1.385 (5)
C2—C3	1.378 (6)		
			
C1—N1—C6	112.0 (3)	N2—C4—C5	121.3 (4)
C1—N1—H1	124.0	N2—C4—C3	119.0 (4)
C6—N1—H1	124.0	C5—C4—C3	119.6 (3)
C4—N2—H2A	120.0	C7—C5—C4	118.5 (3)
C4—N2—H2B	120.0	C7—C5—H5	120.8
H2A—N2—H2B	120.0	C4—C5—H5	120.8
O1—C1—N1	124.6 (4)	O2—C6—N1	124.6 (4)
O1—C1—C8	129.0 (4)	O2—C6—C7	129.8 (4)
N1—C1—C8	106.4 (3)	N1—C6—C7	105.6 (3)
C3—C2—C8	118.8 (4)	C5—C7—C8	121.5 (4)
C3—C2—H2	120.6	C5—C7—C6	130.5 (4)
C8—C2—H2	120.6	C8—C7—C6	108.0 (3)
C2—C3—C4	120.9 (4)	C2—C8—C7	120.7 (3)
C2—C3—H3	119.5	C2—C8—C1	131.3 (4)
C4—C3—H3	119.5	C7—C8—C1	108.0 (4)

### Hydrogen-bond geometry (Å, °)

**Table:**

*D*—H···*A*	*D*—H	H···*A*	*D*···*A*	*D*—H···*A*
N1—H1···O1^i^	0.86	2.09	2.924 (4)	164
N2—H2B···O1^ii^	0.86	2.28	3.122 (5)	167
N2—H2A···O2^iii^	0.86	2.17	2.996 (4)	161

Symmetry codes: (i) −*x*+1, −*y*+1, *z*+1/2; (ii) −*x*+3/2, *y*+1/2, *z*−1/2; (iii) −*x*+1, −*y*+2, *z*−1/2.
